# Oral Care for Children with Special Healthcare Needs in Dentistry: A Literature Review

**DOI:** 10.3390/jcm11195557

**Published:** 2022-09-22

**Authors:** Hamdan Alamri

**Affiliations:** Department of Preventive Dental Sciences, College of Dentistry, Majmaah University, Al-Majmaah 11952, Saudi Arabia; h.alamri@mu.edu.sa ; Tel.: +966-56-375-5537

**Keywords:** disabled children, oral health, dental care, dental care for disabled, access to care

## Abstract

Oral health is a very important aspect of general health, especially for vulnerable groups such as children with special healthcare needs. It is important to provide appropriate oral care in order to promote quality of life and good health for everyone, especially for children with special healthcare needs. Method: We reviewed the recent literature to collect knowledge regarding the delivery of quality oral care to children with special healthcare needs. We also explored some of the treatment management options that could address the needs of these children when attending dental clinics. Result: Unfortunately, we noted significant inequalities with issues related to oral health among those children. This situation often results in limitations to the activities of daily living for these children. There is therefore a need for much-needed advancements and refinements in oral healthcare to address the needs of children who have special healthcare needs. Conclusions: Providing children with special healthcare needs with high-quality dental care may necessitate active liaisons with healthcare facilitators and will require work across professions to make certain that these children’s oral health is also prioritized. Coordinated efforts by dental professionals are needed to provide dental health education and preventive interventions for these children.

## 1. Introduction

Children with special healthcare needs (SHCNs) comprise an expansive section of society, encompassing children living with chronic physical, cognitive, communication and/or behavioural difficulties [1,2]. More than 1 billion people (roughly 15% of the global population) are recorded as having some sort of disability or special need [3]. Of these, there are an estimated 93 million children (aged 0–14) who are living with moderate or severe needs; of these, 13 million children suffer severe difficulties. For people aged 15 or over, around 892 million live with moderate or severe needs, with 175 million living with severe difficulties [3].

According to an estimate of the World Health Organization (WHO), the proportion of the population living with disabilities or special needs in developing countries amounts to 12%, while the same indicator in developed countries stands at 10% [4,5]. There is, however, a deficiency in the calculation of the estimates of the global data when it comes to the prevalence of individuals with special needs or disabilities, and it is thus a matter of urgency that a robust, comparable and comprehensive dataset be collected in this regard [6]. It can be problematic to obtain accurate data on children living with special health needs given the spectrum of conditions that may be included in this classification. The identification and categorization of SHCNs in the majority of developing nations are also hindered by shortcomings in culture- and language-specific instruments for data collection [4,5].

The special healthcare needs of children can arise as a result of intellectual, physical, social or emotional impairment [7,8,9]. Special healthcare needs are defined as:

*“…any physical, developmental, mental, sensory, behavioural, cognitive or emotional impairment or limiting condition that requires medical management, healthcare intervention and/or the use of specialized services or programs. The condition may be congenital, developmenta, or acquired through disease, trauma or environmental cause and may impose limitations in performing daily self-maintenance activities or substantial limitations in a major life activity.”*

[10]

Furthermore, as the population continues to grow, the demand for dental care for individuals with special needs also increases. As Polli et al. (2016) state:

*“Once the expectation of population lifetime has increased, the demand for dental treatment for patients with intellectual disability, physical limitations, social and/or emotional deficit also grew.”*

[11]

Previous studies have reported that the prevalence of dental caries in children with SHCNs is similar to other children of the same age [12]. However, the oral health of children with SHCNs usually deteriorates faster than that of the general population as they grow older. There are fewer restorations, more missing teeth and more untreated dental caries found in children with SHCNs than in the general population [7,13,14]. Moreover, a systematic review carried out by Davis and Anders (2010) emphasised that the prevalence of untreated dental caries and periodontal disease is higher among those with SHCNs compared to the general population [13]. Oral health also has a significant impact on the psychological health of an individual. Poor oral health, for example, can result in reduced nutritional intake, impaired social interactions, difficulty in undertaking day to day activities and associated anxiety [15,16].

The implications of poor oral health are substantial, with some evidence reporting the effects of poor oral health among children with SHCNs on their wider health [17,18]. Recent findings indicate that there are existing health inequalities for children with SHCNs [6,19]. It is imperative that children who are more prone to caries receive preventive dental care [19]. Those with SHCNs in receipt of such care will be more likely to have their oral health needs met [11,20]. Several obstacles to preventive care exist, however, and these can include lack of access to dental care, lack of ability of dental professionals to care for children with SHCNs, lack of cooperation at dental appointments, oral aversions, other overriding medical needs and the financial and psychological burden on the child’s family [9,21,22].

It should also be borne in mind that some children with SHCNs are often unable to grasp the importance of preventive oral health practices and/or will be unable to cooperate accordingly [20]. In cases where the child is very young, where the child has serious conditions or where the child is accommodated in a care facility, the child’s oral hygiene is the responsibility of whoever takes care of them. This can be problematic when a parent or caregiver lacks knowledge and understanding about the significance of oral hygiene [21].

Oral health is an essential part of an individual’s overall well-being. This is particularly true for children with special healthcare needs (SHCNs) as they are at an increased risk of developing oral diseases throughout their lifetime [22,23,24]. Furthermore, children with special healthcare needs have additional oral health requirements that necessitate management in a dental care setting that has been adapted to their specific needs by an oral care provider with specialised knowledge and training. Dental care for children with special needs is still not considered a priority by some in healthcare systems, despite calls for research into the optimum management of these children [25,26]. Wright (1975) argues that it is crucial to invest in creating a positive attitude towards oral health services for children with SHCNs and to involve them in this process to aid ongoing prevention and improve oral health in the future [27].

Thus, despite its clear importance, delivering good oral care to children with SHCNs can be fraught with difficulty [28]. The challenges such children face are constant, and their serious oral needs often go untreated [29]. Moreover, this population’s ability to access necessary dental care can be hindered by the complex nature of their wider medical issues as well as behavioural problems and the family’s involvement in attaining proper oral care [29].

Children with SHCNs should, regardless of difficulty, be able to obtain good oral health, which includes an absence of pain and the capacity to consume and enjoy food [30]. However, the reality is quite different, with their healthy peers usually experiencing a significant advantage in relation to the provision of oral care [31]. Although notable developments have been recorded in dentistry through the decades, the problem of delivering good oral care to children with SHCNs persists [32,33,34,35]. This review aims to collect knowledge regarding issues that could hinder or delay the delivery of appropriate and quality oral care to children with special healthcare needs.

## 2. Materials and Methods

We selected key barriers identified through the literature, and for each barrier, we undertook a search of the existing literature. The search was undertaken by the main re-searcher using MEDLINE and EMBASE with key words such as ‘oral health’, ‘dental care’ and ‘dental care for disabled’. A search of relevant guideline websites was also undertaken (e.g., NICE, SDCEP, RSC, BSDH and AAPD). It was not the purpose of this review to perform a systematic review of all relevant research evidence, but rather to identify synthesised evidence that was most useful for informing the practice/service delivery of oral care to children with special healthcare needs.

The included studies were evidence that met all the following inclusion criteria: literature published in English from 2004 onwards, evidence listed for specific or clinical circumstances or conditions for children and with SHCNs related to oral healthcare, evidence targeting children under the age of 18 with any disability or chronic health condition in dentistry and evidence reported on outcomes related to service access or barriers. Additionally, eight websites for guideline were searched to find guidelines for children with SHCNs in dentistry. One assessor examined and evaluated the titles as well as the abstracts of each of the retrieved records to determine which ones were appropriate and should be selected per the eligibility criteria.

## 3. Results

**Figure 1:**
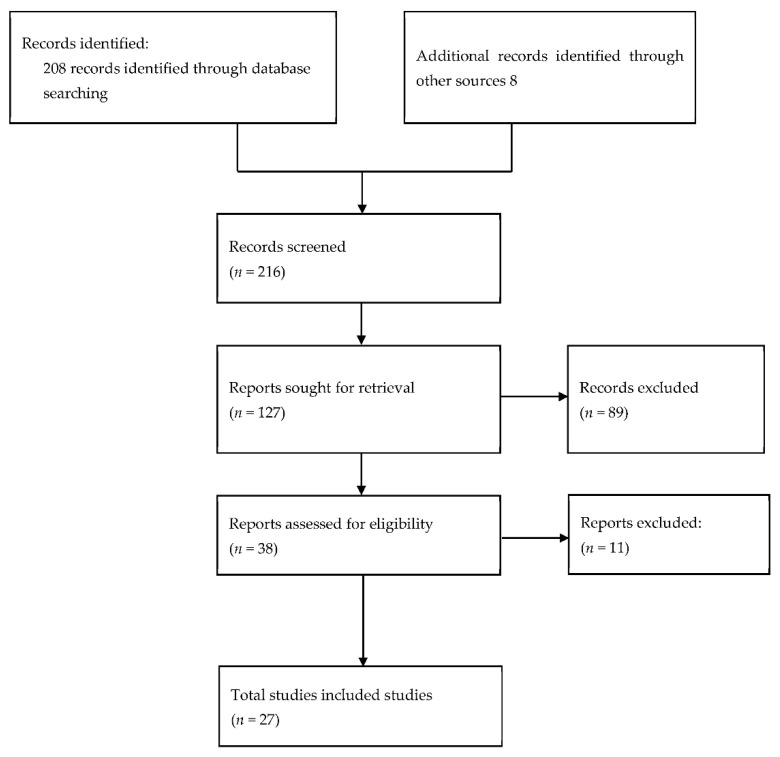
PRISMA flow diagram.

The reviewed evidence focused on the provision of oral healthcare and issues related to it, oral care for children with special healthcare needs, issues related to the delivery of oral care to these children, such as access to dental care services and behaviour challenges both at home and in a clinic. Database searching identified 208 references, with an additional 8 records identified through other sources. From those studies, 27 studies met the inclusion criteria of this review. This process is presented as a flowchart in Figure 1.

This review focused mainly on structural and systemic barriers. Although barriers and access to oral healthcare services are extensively researched topics, the research focusing on oral healthcare issues experienced by children with SHCNs is very limited. This review will discuss its findings accordingly.

### 3.1. Historical Background for Oral Healthcare

Before World War II and immediately thereafter, there was no separate dental care focus or care for children with special needs. The provision of care was widely blended on account of the widespread burden of dental caries, a lack of understanding of the additional healthcare needs of children with infectious and congenital disorders and the considerable lack of knowledge and awareness in specialised dental practices [36].

It was in the late 1950s when awareness emerged, and interest grew amongst dental practitioners to form new approaches towards care for people with additional healthcare needs. It was during the same period that the Academy of Dentistry for the Handicapped was established, which later became known as the Academy of Dentistry for Persons with Disabilities [37]. At the beginning of the 1960s, attention towards the disabled improved significantly. It was during these years that ‘paediatric dentistry’ took an active role in the provision of services along with other rehabilitative and medical practices, including a special focus on the care and requirements for special needs patients [36].

During the 1970s, the Robert Wood Johnson Foundation funded educational courses in dental schools, which focussed on special and disabled patients; this was mainly prompted by the identification and recognition of the dental care needs of the disabled [37]. However, it has been argued that these programmes were only marginally successful, as only a small percentage of graduates of those programs showed an increased acceptance of disabled patients in their clinics. Furthermore, most of the dental institutions that were participating in those courses failed, with only minor improvements and acceptance of these children during the period [37].

In the early 1980s, it became compulsory for British dental schools to integrate appropriate practices and approaches that would meet the requirements of patients with special needs into the curriculum in order to receive accreditation [38]. With this integration, a more complex focus was brought forth in the area of paediatric dental practices for children with special needs. Even though effective reforms were established in dental practices and institutions during this period, progress remained stagnant due to ineffective clinical and curriculum structures [38].

### 3.2. Oral Healthcare of Children with SHCNs

As previously stated, oral health is important for all children and especially for children with SHCNs, who are more vulnerable to oral disease than other children [8,39,40]. Children with SHCNs face many everyday challenges in maintaining good oral health [8,41]. Delivering oral care is crucial for children with SHCNs, although in reality it remains acutely challenging [28]. Children with SHCNs often present with additional challenges both medically and dentally, which can often mean that obtaining appropriate dental care to meet their particular needs is difficult [29].

Delay in tooth eruption is one of the abnormalities that children with SHCNs can encounter in their life; this delay can sometimes even extend to two or three years of age. Another anomaly is when a child has malformed teeth; this may lead to crowding or poor alignment of teeth, which is considered to be a general cause of dental issues such as gum disease and tooth decay [42]. For children with learning disabilities or cerebral palsy, there is a high possibility that they will grind their teeth, which can cause the enamel to break down and cause more issues for dentition [43].

Children with SHCNs resulting from brain injury or genetic conditions can suffer from seizures that put them at increased risk of traumatic dental injury, which requires further assessment and treatment [44]. Furthermore, oral disease can be a consequence of prescribed medications or particular childhood behaviours. Medication with high sugar content is an additional concern, as this will result in increasing the chances of developing new oral diseases or worsening existing oral diseases [45]. Furthermore, medications used to manage seizures can result in gingival overgrowth; other medications such as glycopyrrolate can result in xerostomia, which increases the risk of oral disease [42]. Excessive tooth-grinding habits during self-stimulation in children with special needs can result in further damage to the child’s dentition. It is also pointed out that patients with immune suppression, due to conditions such as leukaemia or any other cancerous or cardiac condition, are at increased risk of various oral health problems [46]. Kokhar et al. (2016) also posit that there is a more significant challenge in the management of oral issues in children with special needs as some of these children lack an understanding of the importance of maintaining good oral health and complying with preventive measures [47].

### 3.3. Issues Relating to Special Healthcare Needs Children (SHCNs)

Dental issues tend to be more prevalent in the children with SHCNs population compared to the general population, and as result, they might require additional preparations to meet their needs [48]. In the UK, the first analysis of the numbers of disabled children with complex needs and life-limiting conditions in over a decade also estimated that numbers increased sharply from 49,300 in 2004 to 73,000 in 2016 [49]. Similar trends can be seen in other countries; data sources from the US National Survey of Children with Special Healthcare Needs indicate that the proportion of children affected increased from 12.8% in 2001 to 15.1% in 2010 [50].

It is estimated that dental care accounts for 8.1% of the unmet needs in children with SHCNs is [48]. Another study suggests that 77.1% of the general population has access to regular dental care and that children with disabilities access dental care significantly less than other children [43]. In addition, another study stated that 14.4% of patients with intellectual disabilities had not received any dental treatment in the preceding 5 years compared to only 8% of the general population [51]. There is less chance of decay being treated in children with learning difficulties, and in cases where treatment is received, the likelihood of extraction is higher [52]. This contributes to the aforementioned poorer outcomes for children with SHCNs, which could thus impact their self-confidence, nutrition, communication and quality of life [53].

### 3.4. Issues in the Home

The presence of special healthcare needs in children may give rise to limitations in performing daily self-maintenance activities to maintain good oral hygiene such as tooth brushing. Due to limitations in their ability to perform oral hygiene activities caused by and motor impairment and sensory and intellectual disabilities, children with SHCNs are prone to poor oral hygiene [29]. Consequently, the oral needs of children with SHCNs require specialised knowledge, increased awareness and attention, adaptation, and accommodative measures beyond what is considered routine [54,55,56]. In one study, the author identifies that the entire challenge extends beyond the children present in the dental chair to the needs of the families as well [36].

Parents of children with SHCNs face many barriers during dental treatment, including, but not limited to, high costs of care and inefficient use of time [30,57]. Children with SHCNs experience higher healthcare utilisation and expenditure than the average paediatric population. Chiri et al. (2012) reported that these children often use more hospital days, emergency room visits, surgical or medical procedures, medical specialist visits, and home health days than children without needs [56]. This extensive use of services may create a financial burden for many families [57].

Klingberg et al. (2012) argue that an additional issue relates to the fact that families usually focus more on the medical treatment of the child rather than his/her oral health [58]. Children with needs may also express greater anxiety about dental treatment than those without a disability, which could delay appropriate dental treatment and lessen their cooperation [59]. All of these barriers can impede the chances of children with SHCNs receiving adequate oral care.

### 3.5. Provision of Oral Healthcare

Parents and caregivers of children living with SHCNs have claimed that healthcare personnel lack the required skills and knowledge to treat them properly. Indeed, a study found that people with special needs, compared to those without, were four times more likely to be frustrated with the care administered and nearly three times more likely to report being overlooked [60]. It was noted by Lindsay et al. (2010) that persons with learning difficulties frequently fail to obtain necessary care [61].

Researchers have reported that some dentists find the delivery of care to children with SHCNs to be excessively difficult and stressful [62]. With this in mind, Dao et al. (2005) indicated that:

*“Health care for individuals with special needs requires specialized knowledge acquired by additional training, as well as increased awareness and attention, adaptation and accommodative measures beyond what are considered routine.”*

[63]

However, certain dentists, whether due to time constraints or perceived insufficient remuneration, are not willing to administer care to such children [6]. Casamassimo (2004) stated that only 1 dentist in every 10 had treated children with SHCNs. As result, patients with special healthcare needs are more inclined to require curative rather than preventive treatment [64].

Smith et al. (2010) found that nearly three-quarters of special healthcare needs patients’ trips to a dental practice were for emergencies and/or extractions. Significant obstacles to the administration of oral healthcare for special needs patients can be exacerbated by the patient’s behaviour, the severity of their existing oral disease and the insufficient knowledge and skill of the dentist (Table 1) [65].

Given the complicated nature of their conditions and their sometimes unpredictable behaviour, a notable proportion of general dentists are neither willing nor suitably trained to deliver care to children with SHCNs [63,64]. Even those dentists who have acquired proper training for special healthcare needs patients often tend to treat only a few children [66]. In addition, Zahra et al. (2018) noted that dental students obtained little training in caring for disabled patients [67]. Meanwhile, Girdler et al. (2009) stressed that effective care for such patients would rely on a dentist’s capacity to control the patient using suitable behavioural management methods, given that such patients, especially those with serious disabilities, may be incapable of cooperating [68].

In addition, the dentist may become a barrier when delivering oral care because of his/her inadequate knowledge and clinical experience. Dao et al. (2005), as well as Waldman and Perlman (2006), state that apart from educational factors several additional non-educational factors, such as adaptations to the clinical environment needed to provide dental care for these patients and concerns about adequate compensation also affect dentists’ willingness to treat special needs patients [64,69]. One study reports that many dentists failed to express an interest in the provision of dental care for children with SHCNs in their clinics, which could be related to a lack of confidence amongst dentists in managing these patients [70]. Furthermore, one of the major problems in treating children with SHCNs is the lack of dental facilities that specialise in treating these children [9].

The number of paediatric dentists who provide care for children with SHCNs is increasing significantly, but despite receiving training in the provision of care for these children, many continue their practice with a standard approach for the care of all children [71]. It is estimated that the total workforce who are trained and skilled in the provision of care to children with SHCNs is far less than what is required, and therefore the capacity to offer dental services to those with SHCNs is extremely inadequate in the current situation [71]. Consequently, the detrimental effects stemming from the current oral healthcare system necessitate analysis and improvement.

### 3.6. Issues with Oral Healthcare Provision

Healthcare infrastructures are frequently under strain from the complex challenges presented by an ever-increasing population with SHCNs [72]. Children with SHCNs tend to visit medical facilities on a more regular basis and for longer periods than their counterparts living without disabilities [73]. The specific service demands in the UK have been outlined in the *National Service Framework for Children, Young People and Maternity Services* [74] and in government policies entitled *Valuing People* [75] and *Together from the Start* [76] (which emphasise the necessity of clear communication and the acknowledgement of how families are affected by caring for children with SHCNs).

An investigation was conducted by Mahon and Kibirge (2004) into the frequency of and reasons behind children with SHCNs being referred to a paediatric assessment unit in the UK from 1997 to 2001. The study showed that children with SHCNs were admitted to a hospital more frequently and for longer periods compared to their peers without needs [73]. Meanwhile, Cooper et al. (2004) asserted that people with special healthcare needs placed a notable burden on healthcare systems [19]. Elsewhere, Newacheck and Inkelas (2004) carried out a study to gather more information about the healthcare experiences of children with SHCNs, revealing they were hospitalised four times more often than their healthy counterparts [77].

In the entirety of dental management literature, there is comparatively little information and data on the current challenges or systematic obstacles to the treatment of children with SHCNs [78]. Another study suggests that five principal issues persist globally in oral healthcare systems for patients with special healthcare needs, irrespective of their age group [79]. These are:

1. Lack of, or sometimes lack of, an integrated delivery system;
2. Lack of academic and regional treatment facilities and institutions;
3. Limited opportunities within a facility to provide interdisciplinary training;
4. The necessity to integrate structured and systemic oral health;
5. The non-awareness of dental caregivers [79].

Stein (2001), in his work ‘*Challenges in long-term healthcare for children*’, asserts profound institutional issues persist in the oral healthcare management of children with SHCNs. He raises not only issues arising from the healthcare system, but also patient issues in the long-term care of children with SHCNs [80]. The entire context, as projected by Stein is shown in Table 1 below. It is important to comprehend the very fact that the difference and the isolated nature of dental practices differentiate them from general health practices. However, in most cases, the standard of practice ethics and rules of dental practice in most countries compel practitioners to attend to the needs of these children (Table 1) [80].

These major obstacles for oral care providers are mainly due to the inadequate system and services available in mainstream dental care facilities to treat these children [36].

### 3.7. Access

In general terms, children with SHCNs suffer from the limited availability of healthcare services [3,81]. Given their special and particular healthcare requirements, they are reported as needing healthcare more than those without conditions [3]. Indeed, studies have revealed that children with SHCNs have more substantial healthcare needs compared to their peers [82]. In a study investigating the quality of hospital care for individuals with learning disabilities in the UK, Brown et al. (2010) determined that certain people with learning needs were not obtaining the right standard of care and that health services were frequently insufficient and unresponsive to meet their needs [83]. Evidence obtained at national and international levels shows that many disabled patients find mainstream services to be inadequate, something that was admitted in a 2012 NHS report [84]. This was reinforced by government demands to make enhanced healthcare for persons with special healthcare needs a top priority [85].

Despite these governmental priorities, children with SHCNs still find it particularly challenging to access oral care services as a result of their complex condition, transportation issues, limited numbers of dentists with the necessary expertise, area of residence and parental education [86,87,88,89,90]. One study reported that up to 25% of parents of children with autism had experienced some difficulty in accessing oral care for their children [88]. In addition, Nelson et al. (2011) reported that 9% of parents and caregivers of children with SHCNs considered it difficult to travel to the oral care provider and that 15% had experienced difficulties in accessing dental care even in clinics close to their area of residence [41].

Accessible dental care presents significant challenges to many children with SHCNs families [62]. Barriers in access to dental care for individual with special needs have also been reported, with transportation difficulties and overall workforce capacity shortages the most commonly encountered obstacles [89]. Nevertheless, dental care is consistently listed as an essential service by parents for their children with disabilities of all ages [78].

It is not only the issues pertaining to the healthcare system that the oral health sector faces, but also the patient-related obstacles or challenges that they may encounter that also affect the entire service. Patient-related challenges include the characteristics that define children with SHCNs and the healthcare delivery system aspects that are ordinarily designed for patients without any disability, which become ineffective in treating the children with SHCNs (Table 2) [36].

### 3.8. Referrals

General dental practitioners in the UK usually provide dental services for children with SHCNs, and they mostly refer those patients with treatment needs beyond their skillset and expertise to special care dentistry or paediatric dentistry services. It is uncommon in the UK for children to have direct access to specialist services unless they are first seen by a primary dental care practitioner [90]. Paediatric dentists typically focus mainly on the treatment of children who require additional care and attention, while special care dentists (SCD) are concerned principally with the improvement of oral health conditions for children with SHCNs. Furthermore, special care dentistry services in the UK are often provided through hospital settings and across the community, which is more convenient for the children [90].

### 3.9. Lack of Specialists

There has been little improvement in the treatment of children with SHCNs in recent years, and therefore, by assessing the current literature relating to dental practise, it becomes clear that only a small percentage of dental practitioners and the associated workforce have made an effort to acquire the awareness and knowledge to treat children with SHCNs. Casamassimo (2014) asserts, “only small percentages of dental practitioners make CSHCN a portion of their practice” [36]. Within this scenario, the majority of paediatric dentists became default practitioners for all children, including children with special needs [91]. For this reason, many paediatric dentists lack the necessary specialisation in treating children with special needs. This results in dentists’ poor communication skills and a lack of knowledge when treating children with uncontrollable movements [92,93].

### 3.10. Lack of Training

Comparatively, the proportion of dental care teams who show an unwillingness to attend patients with special healthcare needs is increasing. One of the main reasons for this unwillingness is the lack of awareness and specialist knowledge to approach patients with special healthcare needs, followed by discomfort from treating patients with special healthcare needs [92,93,94,95,96,97].

These two issues have consistently been asserted in this field for a prolonged period and confirm that ineffective dental education contributes to the community of dental practitioners who are unwilling and unskilled to care for children with SHCNs. Even though there is a significant challenge for parents and dental professionals to manage children with SHCNs, it is also important to signify the inability of these dental professionals to manage these kinds of complex situations [94].

According to Lehl (2016), the main reason behind this inability is the lack of proper knowledge, education, experience, and training [95]. This position was affirmed recently by a study of a large number of ADA members who reported that general dental practitioners are unwilling to treat patients with special healthcare needs, citing a lack of appropriate training, knowledge and awareness [65]. Specifically, Smith et al. (2010) stated that among all the dental schools, only a few provide facilities for activities that focus on the special needs patient, and consequently the lack of willingness on behalf of the practitioner is unsurprising. It is indeed not straightforward to train general dentists in the care of children with SHCNs, and the same is true for paediatric dentists. Meanwhile, it is also important to understand that by providing dental care for the children or patients with special needs, there is no extra financial gain for these teams. This may be one of the main reasons for these professionals to remain unskilled or otherwise choose to be unskilled in these areas. However, Lehl (2016) points out that the experience gained through these situations becomes a long-term asset for the practitioners and professionals in these areas [95]. Patients with special healthcare needs, especially children, sometimes undergo the same procedures as non-special needs patients, but more time and effort need to be given to successfully complete the procedure.

Providing a routine dental assessment can be challenging for dental care professionals for a variety of reasons, including gaps in, or absence of, professional training; limitations in the working environment; difficulty in adaptation due to the change in the environment; the lack of specialist equipment or the lack of proper scientific knowledge relating to the patient and the condition [92]. These factors can occur alone or in combination.

There have been many suggestions made to improve the situation which consider the many past experiences of treating members of such categories. The suggestions include improved training for the undergraduate and the postgraduate dental student, increased access to training for qualified practitioners and discretionary fees to the practitioner to compensate for the additional time required for dental appointments [98]. The needs are currently being addressed by the Teachers Group of the British Society for Disability and Oral Health in the UK, and they have led to new clinical education plans and development programs for undergraduates in the dental unit [52].

Due to the increased healthcare utilisation, many dental schools around the world are enhancing and restructuring their curriculum, and this includes changes to accommodate the dental management of children with SHCNs [36]. A large number of healthcare providers, professionals, policymakers, families and other concerned individuals have enforced the improvement in the oral healthcare system for children with SHCNs and also put in significant effort to ease the challenges in accessing oral health [99,100].

The behaviour management of children with SHCNs is often a challenge to the dental practitioner. Children can display resistant behaviours in different forms as a sign of dental anxiety [100]. Such behaviours can interfere with the delivery of safe and comprehensive dental care. Therefore, parents and caregivers need to be present to allow the practitioner to be able to manage unexpected behaviour in the dental room [10]. Physical stabilisation is another debatable management technique believed by some to be imperative in children whose traditional attitude guidance approaches are inadequate [101]. However, it is recommended nowadays that there should be limited use of this method and only after acclimatisation, distraction and desensitisation strategies have proved to be ineffective [102]. As such, the Department of Health (UK) Guidance for Restrictive Interventions acknowledges the need for such actions as physical restraint in the provision of oral care [103]. As stabilisation during dental treatment has been shown to increase levels of anxiety, dentists must carry out risk and benefit assessments prior to the use of physical restraint [103]. If the stabilisation is not feasible, sedation is an alternative behavioural management technique [104].

Oral healthcare providers may use different techniques for behavioural modification and various pharmacological control strategies in these situations. However, children with greater needs or disability may need general anaesthesia or adjunctive sedation [105,106]. A study on the use of a 50% mixture of nitrous oxide/oxygen was proved to be successful among approximately 92% of those surveyed, with only minor side effects observed among them [81]. Children with SHCNs are among the most frequent individuals for whom an oral care provider uses general anaesthesia [107]. However, the use of general anaesthesia should be the last resort for families of children with SHCNs due to adverse drug interactions, lingering effects and its high cost, which may reduce the frequency of dental visits [108,109].

Overall, children with SHCNs require coordinated care from the caregiver, dental staff and dentist. They also require services of special clinics, programs, and experienced as well as trained personnel. Therefore, the lack of availability of and access to proper oral care for special needs patients is something that must be addressed. Creating greater knowledge as well as skills among the dentists and dental clinic staff members to support children with SHCNs can make access to dental services better. The dental treatment of children who have mild or moderate levels of disability can be carried out in a primary care unit—where their other family members get treated—without much difficulty.

## 4. Conclusions

Children with SHCNs often present with several medical and oral conditions, and the existing lack of understanding from all stakeholders in relation to their specific requirements can interfere with the delivery of optimal oral care. The barriers to the provision of oral care that have been detailed document the fact that it is vitally important to implement measures to ensure that appropriate healthcare facilities are available to children with SHCNs. In addition, dentists should have proper knowledge and training in order to adapt their practices so that the needs of these individuals in terms of oral healthcare can be met. All efforts should be incorporated to support this demographic as different forms of management and treatment approaches become available and can be utilised to allow children with SHCNs to receive the correct and necessary dental treatment required.

## Figures and Tables

**Table 1:** Issues in the system that affect the oral healthcare of CSHCNs.

Issues in the System	Examples Occurring in the Oral Healthcare Delivery System
Dependence on Technology	When the patient is confined to bed or wheelchair When the patient is respiratory dependent Prompting more than one visit, due to gastronomy feeding
Dependence on Caregiver	Oral healthcare and the delivery of home health activities are affected due to the blurring of roles Dire lack of clarity when it comes to consent, payment and other problems mainly related to the care service
Dire lack of proper definition when it comes to oral healthcare	Issues in the reimbursement system and the denial of medical requirements that would affect the correct development and facilitation of rehabilitation Issues pertaining to the focus of oral healthcare sub-departments, such as dietary modification, oral surgery and even sometimes physical therapies
Dire lack of adequate services	Oral health interventions that are under-skilled (for example, healthcare offered by non-dentist practitioners) Insufficient approval towards special oral healthcare practices (for example, general anaesthesia covering the restorative care) Inappropriate services and practices getting approved (for example, paying for lingual frenectomies)
Care in the financing section	Limitation in covering various special requirements Low payment system for various dental procedures in public programs
Care Delivery Models	Institutional dental services that are poor in quality Improper coordination of the dental service with other general health departments Dire necessity in the special oral health expertise Inappropriate transition to adult healthcare
Issues in the quality of the dental service system	Inappropriate or improper quality management system, standards and measures

**Table 2:** Issues relating to special healthcare needs patients [51].

Issue Areas	Examples of the Issues
Accessibility	Institutions that are considered difficult to access physically Institutions that are not situated on the transportation route Institutions that do not have the facility to accommodate special needs, which also creates scheduling problems
Financial	Institutions that do not have special needs medic-claim policies Institutions that are unaware of secondary or alternate funding resources Low-quality training and sometimes underemployment
Psychosocial	Complex health problems Fear to receive or approach healthcare Socially deprived Low intellect Lesser priority in oral health
Stability and Mobility	Movements that are uncontrollable Weaknesses in the muscles Short focus span Hyperactivity
Communication	Speech, sensory or intellectual disability
Medical	Special medication Allergies Atypical cognitive ability

## Data Availability

Not applicable.
